# Ethical issues surrounding the provider initiated opt – Out prenatal HIV screening practice in Sub – Saharan Africa: a literature review

**DOI:** 10.1186/s12910-015-0068-y

**Published:** 2015-10-24

**Authors:** Luchuo Engelbert Bain, Kris Dierickx, Kristien Hens

**Affiliations:** Centre for Population Studies and Health Promotion, CPSHP, BP 7535 Yaounde, Cameroon; Department of Military Health, Ministry of Defense, Yaounde, Cameroon; Interfaculty Centre for Biomedical Ethics and Law, KU Leuven, Belgium

**Keywords:** Ethical aspects, HIV screening, Prenatal, Sub – Saharan Africa, Informed consent, Opt – out, Opt – in, Acceptability and pregnancy

## Abstract

**Background:**

Prevention of mother to child transmission of HIV remains a key public health priority in most developing countries. The provider Initiated Opt – Out Prenatal HIV Screening Approach, recommended by the World Health Organization (WHO) lately has been adopted and translated into policy in most Sub – Saharan African countries. To better ascertain the ethical reasons for or against the use of this approach, we carried out a literature review of the ethics literature.

**Methods:**

Papers published in English and French Languages between 1990 and 2015 from the following data bases were searched: Pubmed, Cochrane literature, Embase, Cinhal, Web of Science and Google Scholar. After screening from 302 identified relevant articles, 21 articles were retained for the critical review.

**Discussion:**

Most authors considered this approach ethically justifiable due to its potential benefits to the mother, foetus and society (Beneficence). The breaching of respect for autonomy was considered acceptable on the grounds of libertarian paternalism. Most authors considered the Opt - Out approach to be less stigmatizing than the Opt - In. The main arguments against the Opt - Out approach were: non respect of patient autonomy, informed consent becoming a meaningless concept and the HIV test becoming compulsory, risk of losing trust in health care providers, neglect of social and psychological implications of doing an HIV test, risk of aggravation of stigma if all tested patients are not properly cared for and neglect of sociocultural peculiarities.

**Conclusions:**

The Opt – Out approach could be counterproductive in case gender sensitive issues within the various sociocultural representations are neglected, and actions to offer holistic care to all women who shall potentially test positive for HIV were not effectively ascertained. The Provider Initiated Opt – Out Prenatal HIV Screening option remains ethically acceptable, but deserves caution, active monitoring and evaluation within the translation of this approach into to practice.

## Background

Significant progress has been made in curbing HIV – AIDS prevalence, incidence and burden in the past two decades. However, over 36 million people still live with the disease worldwide especially in Sub – Saharan Africa [1]. The advent of Highly Active Anti-Retroviral Therapy, (HAART), has drastically changed the course of HIV infected persons. The disease has evolved from being a deadly infectious condition, to a chronic disease. Nowadays, most infected persons live a normal life due to the improved access, availability and effectiveness of Anti – Retroviral Therapy [1, 2]. Testing pregnant women for HIV at the time of labour and delivery is the last opportunity for Prevention of Mother-To-Child HIV transmission (PMTCT) measures, particularly in settings where women do not receive adequate antenatal care. About 60 % of the general population globally remains untested, or is unaware of their HIV status [3]. Despite the availability and effectiveness of new rapid HIV tests and prevention strategies, many women are still seen in labor rooms with uncertain HIV serology status [2, 4]. Tudor Car and colleagues reported in a recent systematic review that over 70 % of women admitted to the labor rooms did not know their HIV status [5].

More than 90 % of the new infections occur during the prenatal period [1]. Over 75 % of perinatal HIV transmission occurs during labor and delivery [6]. If the HIV status of the mother is known especially during the prenatal or intrapartum period, affordable and effective interventions (specific medical staff practices and anti – retroviral therapy) for immediate protection of the neonate and treatment for the mother can be readily provided. Transmission rates from infected mothers to their children could be reduced to 2 % or less with the current Prevention of Mother to Child Transmission of HIV prevention package [4, 6]. Prenatal and labor room HIV screening could be a starting point to get to the sexual partners of infected mothers since some of these mothers and their husbands or sexual partners are generally unaware of their HIV status [2, 5]. The World Health Organization (WHO) in 2003 reported that only half of the pregnant women living with HIV had received Anti Retroviral Therapy for the prevention of Mother – To – Child Transmission of the disease [7]. One third (32 %) of women who give birth in developing countries do so without any previous antenatal follow up [4, 8]. Worldwide, the main approaches that have been used in the prenatal HIV testing services are the Opt – In and Opt – Out approaches.

In the Opt – In approach, the pregnant women are given pre-HIV test counselling, and they actively choose to receive the test usually in writing (written informed consent). With the Opt – Out approach, pregnant women are informed that an HIV test will be included in the standard group of prenatal tests (that is to say, tests given to all pregnant women), and that they may decline the test. A written document is often not required and the HIV test is incorporated into the usual package of routine care tests. Unless they decline, they will receive an HIV test [9, 10]. However, some authors have questioned recently if the opt -out approach is actually justified from an ethical perspective [11–15].

In most Sub – Saharan African countries today, the main approach to HIV screening in prenatal care settings is Provider Initiated Testing and Counselling (PITC) using an Opt - Out approach. Some authors have argued that women from this region of the world undertake the HIV test under some degree of implicit or “involuntary” coercion, and that the consequences when these women would realize that they actually had a choice either to turn down or to comply with the test could be counterproductive [15–19]. These consequences could range from lack of trust in the health care providers to refusal to take part in future research activities [20], [19]. To better appreciate whether this prenatal HIV screening approach is appropriate or not from an ethical perspective, we therefore carried out a review of the literature using a reasons based approach [21, 22]. The main objective of this study was to ascertain the ethical arguments for or against the provider initiated opt – out approach in prenatal HIV screening within the Sub – Saharan African context.

## Methods

Multiple and at times potentially conflicting interests are at stake when it comes to testing the pregnant woman for HIV: the interests of the pregnant woman, the fetus and the interest of society [7, 9, 23]. The goal of this review was to identify the main ethical arguments that have been put forward in the literature in support of or against the provider initiated opt – out prenatal HIV screening approach within the Sub – Saharan African context. Strech and Sofaer have argued that argument – based literature reviews are valuable tools that can be used to improve ethically relevant decisions in healthcare, research or policy [21]. McCullough et al. [22] support the use of argument – based literature in bioethics and propose a four step methodological framework in performing such a task. The four step methodological framework proposed by McCullough et al. in conducting reviews of argument - based literature was used as the main construct in answering our main research question of whether the current provider initiated opt – out prenatal HIV screening approach in Sub – Saharan Africa is ethically justified or not. These steps were:

I. Identifying a focused question: Is the Opt – Out approach in prenatal HIV screening ethically justified in Sub – Saharan Africa?
II. Conducting the literature search with specific key words.
III. Assessing the adequacy of the arguments for and against this approach
IV. Identifying conclusions drawn in each paper to assess whether they apply to the identified focused question.

We searched the following data bases: Pubmed, Cochrane literature, Embase, Cinhal, Web of Science and Google Scholar. To carry out a search, the following search terms were employed: Ethical aspects, HIV screening, Prenatal, Sub – Saharan Africa, Informed consent, Opt – out, Opt – in, acceptability and Pregnancy. All articles published in English and French between 1990 and December 2015 that were directly related to our research question were carefully reviewed. Articles which were not related to ethical issues with regards to HIV screening in pregnancy were excluded. Reference lists of selected articles were carefully checked through snowballing to identify other key related articles or documents of interest.

A total of 302 articles were identified. Article duplicates were removed. Editorials, policy papers and commentaries were not included for analysis. After screening, 21 of these were included for an in depth review. All of the selected articles were published in English. The main research question that guided the review and analysis process was: What are the main ethical arguments in favor of or against the Provider Initiated Opt – Out Prenatal HIV screening approach in Sub – Saharan Africa?

## Results (The main findings are summarized in Table 1)

**Table 1:** Ethical arguments for or against the Provider Initiated Opt – Out Prenatal HIV Screening

Title	Author(s)	Year of Publication	Main Themes
1. Legal and Ethical Implications of Opt-Out HIV Testing	Hanssens C [13].	2007	- Rigid application could trigger legal claims - Risk of breaching informed consent, - Disregard or emotional and mental risks - Could disregard autonomy and dignity
2. Desperately seeking targets: the ethics of routine HIV testing in low-income countries.	Rennie and Behets [40].	2005	- Cannot be effective since clients are generally unaware of the meaning of the opt –out approach - Conflict /balance between promoting good (conscious and unconscious coercion from training of health care staff and insistence on right to refuse test (wrong message that test is unimportant). - Reduction of HIV – AIDS related stigma - Not adapted to areas characterized by high poverty levels, gender inequalities, weak health care infrastructure and poor access to treatment
3. HIV testing of pregnant women: an ethical analysis.	Johansson et al. [12]	2011	- Most effective strategy - Recognize flip side of the strategy becoming involuntary in the clinical setting - Availability and effectiveness of inexpensive drugs makes intrusiveness of test less important that hypothetical preference of the child to be born healthy.
4. The Uptake of Integrated Perinatal Prevention of Mother-to-Child HIV Transmission Programs in Low- and Middle-Income Countries: A Systematic Review.	Tudor Car et al. [5]	2013	- 11 % of women delivered in labor rooms do not know their HIV results and did not participate in any HIV prevention programme - High uptake of the Provider Initiated Opt – Out prenatal screening approach (96 %) - Retention in Prevention of Mother to Child Transmission of HIV Interventions low (17 % of Antenatal care attendees).
5. Routine offer of antenatal HIV testing (“opt-out” approach) to prevent mother-to-child transmission of HIV in urban Zimbabwe.	Chandisarewa et al. [11]	2007	- More mother/infant pairs receive treatment when screened using the opt – out approach - Greater compliance with the opt – out screening approach - Greater reported satisfaction with this approach compared to the opt in approach - Lower reported rates of spousal abuse
6. Consent and antenatal HIV testing: the limits of choice and issues of consent in HIV and AIDS	Sherr at al [14].	2000	- Poor information transmission from health care providers to pregnant women with regards to HIV transmission risk and obtaining consent in antenatal settings could be counterproductive in terms of: a. Test uptake b. Optimal goal of minimizing maternal/fetal transmission.
7. Could you have said no? A mixed-methods investigation of consent to HIV tests in four African countries.	Obermeyer et al. [16]	2014	- Underscores HIV testing with current opt – out approach without any consent from pregnant women (7 %) - Health care providers actively influence the choices of clients (21 %) - Quality of questions asked to evaluate the informed consent procedure is of utmost importance - Retrospectively asking clients if they would have said no overestimates measured levels of coercion
8. Rethinking HIV exceptionalism: the ethics of opt-out HIV testing in sub-Saharan Africa.	April [18].	2010	- Opt – Out testing could increase survival only with effective case management and sustainability plans - Opt – out screening encourages test acceptance, while opt – in screening could increase test refusal rates - Opt – out screening restricts autonomy, but justifiable on ethical grounds based on the theory of libertarian paternalism
9. Opt-out HIV testing: an ethical analysis of women’s reproductive rights.	Fields and Kaplan [23].	2011	- Screening approach is at odds with true informed consent - Reproductive rights principles risk being disregarded if counseling is not properly done - Increases testing rates and permits appropriate reproductive choices to be made by mothers - Suggests training of health care staff to offer ethically acceptable counseling and testing, as well as societal actions to decrease HIV associated stigma and discrimination
10. The sexual ethics of HIV testing and the rights and responsibilities of partners.	Dixon-Mueller [54].	2007	- Rights of individuals to refuse testing ignores the rights of other sexual partners to be informed of the health risks they are exposed to. - With the Opt – out testing approach, breaching autonomy could also go against the right not to know of the clients. - Opt – out approach justified on grounds of doing no harm to the fetus
11. Program synergies and social relations: implications of integrating HIV testing and counselling into maternal health care on care seeking.	An et al. [36]	2015	- Integration of HIV testing with routine care improves confidentiality, more convenient and less stigmatizing - Opt – out HIV screening within the prenatal care package is perceived as compulsory - Lack of trust in health care staff and non – supportive health care provider – client relationships aggravates stigma and favors loss to follow up. - Social relationships between clients and health care staff must be well understood and acted upon for this approach to be ethically acceptable
12. Is ‘Opt-Out HIV Testing’ a real option among pregnant women in rural districts in Kenya?	Ujiji et al. [15]	2011	- HIV test is considered compulsory by most clients with only 17 % thinking it is optional. - High coverage of HIV testing appears to be achieved at the cost of pregnant women not understanding that testing is optional
13. Routine antenatal HIV testing: the responses and perceptions of pregnant women and the viability of informed consent. A qualitative study.	De Zulueta and Boulton [25].	2007	- No pregnant woman tested fulfilled the standard criteria for informed consent - Routine testing (Opt – Out) compromises informed consent - Almost no understanding or false beliefs with regards to a positive HIV test result amongst most participants
14. An offer you can’t refuse? Provider-initiated HIV testing in antenatal clinics in rural Malawi.	Angotti et al. [17]	2011	- An HIV test is a compulsory precondition to receive antenatal care - Benefits of antenatal HIV testing are more important than choice - People may increasingly avoid government hospitals for antenatal services to escape what they perceive to be a mandatory testing requirement.
15. Rethinking mandatory HIV testing during pregnancy in areas with high HIV prevalence rates: ethical and policy issues.	Schuklenk and Kleinsmidt [56].	2007	- Prevalence rates of HIV in test refusers generally greater compared to accepters. - Argue from a purely consequentialist perspective: stress of subjecting clients to mandatory test is far less (outweighed) by the benefits of knowing infected mothers status and implementing appropriate prevention and treatment strategies - Provide four preconditions for mandatory screening in high HIV prevalence settings: woman voluntarily decided to carry fetus to term, have reasonable alternative courses of action (eg abortions), available and voluntary HAART treatment and confidentiality should be guaranteed.
16. The complexity of consent: women’s experiences testing for HIV at an antenatal clinic in Durban, South Africa.	Groves et al. [47]	2010	- Generally, some women have a clear choice to get tested, others not very sure and others feel they have no choice. - Direct and indirect coercive techniques employed by heath care staff to get them tested. - Further studies to develop mechanisms to simultaneously meet up with public health goals of widespread test coverage while respecting women’s autonomy.
17. From caution to urgency: the evolution of HIV testing and counselling in Africa.	Baggaley et al. [45]	2012	- Provider Initiated Testing and counseling generally acceptable throughout Sub – Saharan Africa - Women generally not aware of the fact that they can decline an HIV test
18. Practicing provider-initiated HIV testing in high prevalence settings: consent concerns and missed preventive opportunities.	Njeru et al. [48]	2011	- Limited pre and post - test counselling with the provider initiated opt – out approach. - Relative neglect on insistence on preventive measures - Clients frustrated with inability to opt – out - The opt – out model in prenatal settings deserves to be revisited and acted upon to maximize protection of client autonomy and access to effective prevention practices
19. Opt-out HIV testing during antenatal care: experiences of pregnant women in rural Uganda.	Larsson et al. [39]	2012	- Clients consider test as compulsory - Benefits of getting an HIV status result not fully discussed to clients and not understood by clients - Compulsory testing could deter clients from seeking antenatal care - Gender sensitive models need to be recognized to encourage partner screening
20. HIV/AIDS Stigma and Refusal of HIV Testing Among Pregnant Women in Rural Kenya:	Turan et al. [61]	2011	- Women who recognize getting stigmatized if partners know their results are more likely to refuse an HIV test, or be compliant to treatment - Anticipated stigma could be barriers to accepting HIV screening by pregnant women - The Opt – Out approach if compulsory without taking into account this reality could aggravate stigma and thus become counterproductive.
21. An Ethical Analysis of Opt-Out HIV Screening for Pregnant Women	Wocial and Cox [27].	2007	- Opt – out approach justified on the basis of the potential good to the general public (beneficence). - Respects autonomy since it is not a discriminatory process - Everyone is screened without discrimination nor neglect of marginalized individuals (justice). - Effective counseling empowers women to take more rational decisions and reduce transmission to their infants - Opt – Out approach considered to be ethically defensible, especially if counseling of women is effectively acted upon.

After abstracting the data from selected papers, four main themes emerged. These were: respect for client autonomy, beneficence, stigma related issues, coverage and acceptability of the HIV test.

### Autonomy

The principle of respect for autonomy remains the main recognized and vulgarized principles since the birth of bioethics. The three main properties of autonomous decisions include [24]:

I. its intentionality and self – direction
II. adequate understanding of information provided and
III. Absence of coercion.

Health care professionals have the responsibility to respect, protect and enhance patients’ freedom of choice [24]. The quantity and quality of the information provided to the clients if inappropriate can jeopardize the respect of autonomy with the Opt - Out screening approach especially amongst pregnant women [12, 16, 25, 26]. The principle of respect for autonomy remains the main ethical principle that can easily be breached with the current provider initiated opt – out screening approach [18, 23, 24]. The theory of libertarian paternalism has been advocated by some authors, to justify at times a more coercive testing approach like HIV in pregnancy [12, 13, 18, 27]. They argue that the incapacity of individuals at times to make decisions that maximize their own welfare and the protection of the good of third parties especially in infectious disease contexts, make some degree of extra compulsion ethically justifiable [28, 29]. The Opt –Out approach is framed to encourage person to make the “correct” choice in accepting the HIV test, while still preserving their freedom to decline the test [28, 29]. There exists a persistent ethical tension between the goal of public health to promote the wellbeing of the mother, the child and society (knowing her HIV status, providing appropriate care, prevent HIV transmission from mother to child, preventing transmission to third parties and partner screening) and the at the same time respecting the mother’s autonomy [12, 16, 23]. The current opt – out approach could be considered an ethically legitimate nudge, that provides a novel paradigm for informed consent with maintenance of mother’s ability to turn down the test if she decides to do so [12, 27]. Cohen is convinced that nudging approaches have the potential to overcome the ethical dilemmas that exist between paternalistic beneficence and respect for patient autonomy [29]. This could be considered ideally ethical if [18]:

I. the clients fully understand the information given them
II. the information is even given them in the first place
III. they are fully aware of the fact that they have the right to turn down the test
IV. the decision to accept or turn down the test is void of all sorts of coercion.

Fulfilling the conditions for a proper counselling could be very challenging, and most of the times unrealistic. Improvement in the quality of antenatal care services offered could permit HIV counselling and screening under this approach to more or less respect the autonomy of the clients.

In the Provider Initiated Opt – Out screening approach, a relatively small amount of time is spent between the health care provider and the client for counselling [9, 10]. Although this may be considered a drawback, Wanyenze et al. argue that abbreviated counselling if properly carried out produces similar, and at times better levels of understanding amongst clients [30]. Although with an inherent opportunity for the client to turn down the test if she so wishes, this approach will be ethically justified on grounds that the client receives the required and complete information package [9, 10, 27], that the health care provider has the required expertize in communicating appropriately generally within a limited amount of time and that the health provider herself has the required knowledge. With these preconditions generally unmet in most health care settings, the question of how informed the informed consent is under this approach remains troublesome. Obermeyer et al. in a study in four African countries (Burkina Faso, Kenya, Malawi and Uganda) reported that 7 % of clients were tested without any consent. With this provider initiated opt out HIV screening strategy, many clients from most recent studies in Sub – Saharan Africa have reported that they were not aware they could have an opportunity to decline the test if they wanted to and that they were coerced to undertake the test as a precondition to continue receiving health care in their respective health facilities [16, 25, 26, 31, 32].

Another argument in favour of the Provider Initiated Opt – Out approach has been that the extensive pre-test counselling and written consent that were inherent to the opt – in approach and voluntary testing and counselling deterred clients from being tested [33]. Hanssens has highlighted that this approach might not appropriately take into consideration consequences like labelling, psychological distress and post-test discrimination by health care providers with patients that turn out to be HIV positive [13].

### Beneficence

Most authors argue that knowing one’s HIV status permits a timely initiation of appropriate therapies, generally affordable and effective today to decrease HIV associated morbidity and mortality [2, 4]. Provision of treatment today is amongst the most important benefits if getting an HIV test done [2, 18]. This is especially important for pregnant women as this could help them in making informed reproductive choices [23]. Liddicoat et al. found in a retrospective analysis of the charts of 221 HIV positive patients who had encountered a health care provider in the previous year, that the risk and need to carry out an HIV test amongst these clients were not properly addressed and the HIV test was only proposed to 27 % of these persons [34]. This suggests that when the responsibility is left in the hands of health care providers to recommend an HIV test on a case by case basis, more missed HIV test opportunities can be recorded [23]. Knowing one’s status ideally enables early initiation of treatment, which reduces the spread of the infection [2]. Massive detection might require health systems to be prepared to treat all HIV infected mothers. However, it might be difficult to ascertain if health systems in Sub – Saharan Africa could accommodate and appropriately manage larger numbers of pregnant women infected with HIV. April [18], Wocial and Cox [27] have proposed that the sustainability and guaranteeing of proper medical care to all women who shall test positive as recommended by the WHO [9] need to be properly evaluated before vulgarizing the Opt – Out screening practice in high HIV prevalence settings like Sub – Saharan Africa. If not, the negative social and psychological consequences that could arise might instead outweigh the benefits from knowing their HIV status (e.g. stigma, social exclusion).

### Stigma

The Provider Initiated Opt –Out prenatal screening approach has been embraced as a less stigmatizing approach, compared to the usual voluntary testing and counseling or opt – in approaches [9, 23, 33, 35]. The reason given for this is that, as part of routine medical care, HIV becomes treated like all other “normal” or usual diseases [9, 10]. However, some authors fear that vulgarizing prenatal HIV screening as with the Opt – Out approach if not well considered could worsen HIV associated stigma [18, 27, 35, 36]. Inadequate and inappropriate counseling, disregard of sociocultural peculiarities and lack of sustainability of the medical care package before and after the test have been identified as key action areas deserving amelioration. In Sub – Saharan Africa where gender bias and HIV associated stigma are still serious concerns, expanded HIV testing risks resulting in other consequences like discrimination, domestic violence and abandonment [37–39]. Csete et al. have proposed incorporation of measures to fight against gender violence and abuse within the package of the Opt – Out screening approach [37]. These include: emergency helplines, training of police and social service staff on issues of AIDS related violence against women, criminalization of marital rape and school based awareness programmes for girls and boys. Rennie and Behets have argued that in settings characterized by extreme poverty, weak health care and civil society capacities, gender inequality and high levels of stigma against HIV – AIDS patients, the Opt – Out HIV screening approach might divert from its initial human rights motivations of offering universal access to HIV – AIDS management [40]. They recommend continuous empirical research to assess the effects of this approach on individuals and communities, from both ethical and human rights perspective, especially when implemented in resource limited settings.

### Coverage and acceptability

The Provider Initiated Opt – Out Prenatal HIV Screening approach has been reported to be associated with an increase in the uptake of the HIV test [9, 41–44]. Research from most studies in Sub Saharan Africa have described this approach is being generally more acceptable to clients compared to the former Opt - In approach. The acceptability rates range from 80 % to 97 % in most prenatal care clinics [11, 12, 41–45]. With the voluntary counselling and testing approach (Opt – In), acceptability rates have generally been low rarely going over 80 % [2, 9]. True and informed acceptability of the opt - out approach has however been put to question [14, 15, 17, 25]. Empirical research findings have been increasingly revealing that the HIV test options presented to pregnant women are biased: no true voluntary options to accept or turn down the test, most clients directly or indirectly coerced to take the test and the quality of the informed consent process described generally as poor [17, 25, 46], 41]. Most clients have reported that the test was presented to them as compulsory by health care providers and many did not know they had an option to turn down the test if they wanted to [39, 46–49]. Johansson et al. also highlight the risk of the Opt - Out approach becoming an involuntary testing strategy if implemented in a real setting without caution [12]. Larsson et al. have recommended active monitoring and evaluation as key components of the Opt – Out prenatal HIV screening practice [39].

## Discussion

The aim of this review of the argument – based literature was to identify ethical arguments pro and against the Provider Initiated Opt – Out Prenatal HIV screening approach in Sub – Saharan Africa. The World Health Organization (WHO) has specified the minimum package of information to be given to clients during the informed consent process including the tests clinical and prevention benefits, the right to refuse the test, the follow – up services that will be offered and the need to anticipate informing any contacts at risk in case the test turns out positive [9]. These conditions are rarely met in most health care settings. Even in developed countries, many women are still seen in labor rooms with unknown HIV serostatus. For instance, Siemieniuk et al. reported cases from Alberta in Canada who were diagnosed either in the labour room or postpartum [20]. About 32 % of women who give birth in Sub – Saharan Africa do so without ever receiving any prenatal care [4, 8]. The Opt out prenatal screening approach should therefore be coupled with increased efforts to improve prenatal care coverage, which has improved, but remains below expectations in this region of the world. The risk of rendering health care professionals negligent and not able to provide the necessary information as required is a major risk of this approach, especially in a region of the world with very limited human resources in health care. Wanyenze et al. have however proposed that abbreviated counselling models with the Opt – Out approach, if well formulated and implemented could produce similar, and even better results compared to the voluntary time consuming standard HIV counselling and testing approaches [30]. This could be worth experimenting in a Sub – Saharan African context, as this region is seriously challenged with scarcity in health care professionals.

### Informed consent, for whom?

For the health care staff or for the patient’s? Informed consent remains a crucial requirement in both healthcare and research settings. However, many scholars have stated that fulfilling its requirements is unrealistic and even mythical [50, 51]. Even in developed countries with generally higher levels of education and more information, most of the patients never fully understand the informed consent form that they do sign [36, 52]. Without written consent, the likelihood that the test becomes compulsory increases [12, 53]. However, Bayer has argued that making it more difficult to say no with regards to taking an HIV test within the opt – out approach can be justified from a public health perspective. He is particularly concerned with the opportunities accrued to treating the infected on time, avoiding treatable and preventable opportunistic infections and reducing transmission to their partners [53]. Dixon-Mueller supports making it more difficult for persons to say no to getting tested from a rights based approach [54]. She argues that labelling the provider initiated opt – out approach as disempowering since some clients may not feel free to say no undermines these same people’s rights to know their HIV status and to make sexual and reproductive decisions based on this information. In settings where the levels of education and awareness with regards to human rights are generally lower, this becomes a serious ethical concern to be tackled more carefully. With this screening approach, clients have been reported to be totally unaware that they were tested for HIV or been directly or indirectly coerced to get tested [16, 17]. Being unaware of an existing option to turn down the test could reduce the provider initiated opt – out HIV screening approach to compulsory HIV screening [23, 27].

In the United Kingdom, Sheer et al. reported over 18 % of the tested pregnant women for HIV believing they could not refuse the test [14]. Njeru et al. have reported limited pre and posttest counseling in Kenya, Tanzania and Zambia as well as neglect of preventive measures with the Provider Initiated Opt – Out HIV screening approach [48]. Considering that these are high HIV prevalence settings, the intended benefits of expanded screening could be blurred by consequences that could result from coercion, test without consent, aggravated stigma, distrust in healthcare staff and non – compliance to recommended treatment regimens even amongst those tested positive [14, 16, 18, 19, 39, 55]. Fields and Kaplan have argued that protecting the best interest of the fetus and enhancing the general good for society (beneficence) through excessive “paternalistic” HIV screening of pregnant woman is ethically unacceptable [23]. They propose a health care model with health care staff being committed to provide the best available care as well as respecting as much as possible the autonomy of the patients. On the other hand, Johansson et al. think that despite the fact that the Opt –Out strategy might become involuntary in the clinical setting, it remains acceptable [12]. They base their arguments on the fact that the increased availability of very effective and inexpensive lifesaving drugs renders ethical concerns raised by an intrusive HIV testing practice less important compared to the child’s hypothetical preferences to be born healthy [12]. Effective drugs for the management of HIV are generally effective, available and affordable in most Sub – Saharan African countries today [2, 4]. Schuklenk and Kleinsmidt are comfortable with the Opt – Out screening approach even if it could turn out to be compulsory HIV screening especially in areas with high prevalence rates like in Sub – Saharan Africa [56]. They however provide key preconditions for this compulsory testing to be ethically justified. These are:

I. the women in question would have had voluntarily chosen to carry the fetus to term
II. they would have had a reasonable alternative to this course of action (e.g., abortion at least until the point of fetal viability)
III. continuing voluntary treatment with HAART would be available to them
IV. The confidentiality of the women’s HIV status should ideally be maintained during as well as after their pregnancy (not imperative).

The ethical framework driving their reasoning however was purely consequentialist in nature [56]. They fail to take into account possible serious psychological consequences (stigma and social exclusion) and confidentiality issues could arise if the HIV test becomes practically compulsory in prenatal care settings [18, 27, 37]. The Provider Initiated Opt – Out Prenatal HIV Screening approach runs these risks if it is not implemented with caution.

Empirical research in bioethics has been embraced and its importance has generally been well recognized [57, 58]. Despite concerns raised by empirical research about the disregard or inappropriate practice of the informed consent procedure, coercion or compulsory testing with this testing approach,[15–17, 25, 26, 31], it might be of interest for public health actors to embrace these claims with caution [16, 57, 58]. As Obermeyer et al. have reported in a study from four African countries that based on the type of question asked, coercion can be overestimated especially when the women are questioned retrospectively [16]. With the closed question “could you have said no”, which has generally been used in most empirical research endeavors to evaluate the quality of informed consent, coercion was overestimated at 77 %. In depth analysis however revealed that 60 % of these women actually consented to get tested.

### In the quest for efficiency or adequacy?

Efficiency must be clearly differentiated from adequacy. Considerations regarding the former most of the time compels health care staff to offer the HIV test simply as part of their job, or to take specific precautions not to get infected when caring for the pregnant women during delivery. Under such circumstances, the responsibility of health care staff as moral agents is eroded [27]. From an ethical perspective, the concept of adequacy possesses an internal morality and should normally generate a moral impulse that the patient receives during the informed consent process. Sherr et al. have underscored the importance of the quality of information given to pregnant women in antenatal clinics and that of a proper process of obtaining informed consent [14]. If not appropriately acted upon, they highlight how counterproductive this could turn out by reducing the HIV test uptake rates and consequently decreasing the chances for preventing the transmission of HIV from the mother to the child. Eyal has argued this constitutes the bases of trust which is key for compliance to proposed behavioral and biomedical regimes in case the mother is infected [19]. He also argues that Coercion, deception, manipulation and other violations of standard informed consent requirements seriously jeopardize the trust, which remains a key requirement for people to seek medical advice, comply with it, and participate in medical research. The success of the Highly Active Anti-Retroviral Therapy used today in managing patients with HIV – AIDS is almost dependent on compliance [2]. Non – compliance does not only lead to poor outcomes in persons infected, but could also predispose to the development and spread of drug resistant strains of the virus. This is already a growing challenge for public health actors in this region of the world [2, 59, 60]. It is rare and most of the time almost inexistent to find skilled staff (Clinical/psychologists, counselors and social workers) being implicated in the diagnosis and care process of mothers infected with HIV. Despite the reported decrease in levels of stigma associated with HIV – AIDS, the condition still remains a very stigmatization condition and it shall take time for this to reduce to expected and enviable levels [1, 2, 9]. Adequate HIV counseling and screening could guarantee a better understanding of the prevention and treatment options, their effectiveness, available alternatives, dispel false beliefs (reduce stigma) and strengthen trust between the health care staff and society [19]. Challenging the knowledge and beliefs of clients with regards to HIV is a sign of respect for her and renders her decisions to be truly well informed (strengthening her autonomy) [24].

Chandisarewa et al. have reported more infant/mother pairs receiving antiretroviral prophylaxis and follow up in clinics with the opt – out approach compared to the opt – in screening approach (*p* <0.01) [11]. Tudor Car et al. have reported in a recent systematic review a high uptake (96 %) of the current opt – out HIV screening approach in prenatal care services [5]. They also paradoxically report a less than expected (17 %) retention rate on current HIV prevention programs of the infected mothers and children at risk. This could be indicative of the fact the testing is not done under optimal conditions to ensure compliance to the available HIV management protocols in this population. There is some skepticism of an inappropriate application of this approach which can instead deter clients from seeking care and lead to loss to follow up [19, 55]. Becker and collaborators have argued that uncoordinated and non-comprehensive aggressive testing could lead to stigmatization and could deter patients from meeting health care providers for the management, not only of HIV, but other diseases [55]. De Zulueta and Boulton have also reported how health care staff indirectly coerce clients to accept HIV testing. In their study, only two out of thirty-two (32) women were aware of the possibility of a woman, HIV positive, giving birth to an uninfected child [46]. This could depict some degree of lack of information and inadequate counseling skills on the part of the health care staff, or purposeful selective retention of certain pieces of information to coerce the pregnant women to accept the test [46].

HIV with the growing availability and affordability of the treatment for sure has become a chronic condition with most persons leading a normal quality of life as the uninfected [2]. However, we argue that the most important purpose of getting an HIV test is not getting to know one’s status. The behavior after obtaining the test results remains capital for prevention and care purpose. An HIV test done under inappropriate conditions could be a guarantee for clients to flee from coming for follow up after results, many not even turning up for the test results and non – compliance [19, 26]. Sub Saharan Africa is facing a growing epidemic of non – compliance to HAART in most areas and associated HIV resistance [59, 60]. April suggests that the potential gains of this approach if appropriate measures to fight against loss to follow up of patient tested positive are not taken will be negligible [18]. He thinks that the lack of mechanisms to track, educate and monitor diagnosed patients risk eroding the expected gains from the Opt – Out HIV screening approach. As the world and scientific community enviably awaits a vaccine or complete cure medications for HIV, securing the effectiveness of available medications through avoidance of non-adherence to treatment and drug resistance remains critical. The quality of the counseling, consent and screening process could be useful avenues to enhance healthy health care provider – client relationships, foster trust and consequently posttest compliance to recommended treatment (prevention) options [19, 40, 55].

The Provider Initiated Opt – Out Prenatal HIV Screening approach might not necessarily clash with client autonomy if appropriately implemented. From a public health perspective, good quality counseling (void of direct or indirect coercion) using an appropriate, pretested and validated abbreviated model could still allow pregnant women to voluntarily decide on whether to take the test or not. More women will come to know their status, receive early treatment and appropriate interventions to reduce the transmission of HIV from mother to child. It is unclear if the Opt – In approach actually enhances the autonomy of the tested parties, since clients at times still feel pressured to undertake the test [9, 18, 27]. Anticipated stigma and discrimination if one turn’s down the test could deter clients from taking the test [61]. An atmosphere of trust and confidence remains a key determinant of test uptake and compliance to evidence based recommendations given to these patients by health care providers. Third party interests in infectious disease scenarios might compel some degree of compulsion to protect third parties. With HIV in pregnancy, it might be argued that some degree of soft compulsion (soft paternalism) is ethically justified to protect the interest of the baby who has a high potential of being born HIV free today.

## Conclusion

The Opt – Out HIV screening approach in prenatal settings has been generally justified on the grounds of beneficence, reduced HIV associated stigma and increased uptake. Irrespective of the fact that the benefits that could arise from knowing the HIV status of pregnant women with this approach could outweigh potential psychological and social harm of knowing one’s HIV status, this does not in any way relief healthcare providers of their responsibility to properly educate and counsel women [27]. The breaching of respect for autonomy has been considered generally acceptable based on the concept of libertarian paternalism, with reference made to its overwhelming potential benefits to the individual himself, the unborn child and the society. The quality of counseling and the informed consent process has generally been reported as being poor or at times nonexistent. It is difficult therefore to directly associate the reported high acceptability rates of the test to voluntariness. Despite the increase in the number of patients that are being screened with the current provider initiated opt out HIV screening strategy, the conditions under which the screening is done remains key and deserves proper research and action from policy makers. It is unclear whether health systems can effectively manage the growing number of patients tested positive with this expanded screening approach as recommended by the WHO guidelines. If not, is it ethically justifiable to get persons come to know they are HIV positive despite the unavailability of an acceptable posttest management package? The potential gains of knowing one’s HIV status are plausible from the prevention and treatment arms, but need to be carefully balanced with the often ignored psychological and social consequences of letting known HIV positive clients go without treatment. Knowing one’s status already is an initial step to intervene to reduce passing the infection from mother to child, and also spreading the virus to third parties. Improving the quality of counseling and psychosocial support staff within the clinical setting in Sub Saharan Africa remains critical. Adequate training of staff specialized in dealing with psychological components of HIV and increase in training of counselors in this domain could be a good beginning. Adequacy of the screening and not mere attaining of targets could be of interest to public health. If not appropriately worked upon, stigma and non-compliance to prevention and treatment protocols might persist. This could result in more infections than expected, high mortality rates, continuous disease transmission “in the shadows” when persons shy away from getting tested and resistance to the current effective biomedical regimes. Efforts to maintain the moral intention of informed consent to protecting the interest of the patient must be reemphasized. If not adequately acted upon, not only shall non – compliance to biomedical treatment regimens for HIV increase with accompanying increase in HIV resistant strains, public health risks experiencing a loss in public trust in health systems and an accompanying decrease in the willingness to use HIV prevention/treatment services and participate in future research. This might not be of any good to the health care systems especially when the “abused clients” come to better understand their rights in the near future.
